# Multilingualism in mental health care – a long way to professionalism

**DOI:** 10.3389/fpsyt.2026.1876785

**Published:** 2026-08-19

**Authors:** Mike Mösko

**Affiliations:** Department of Applied Human Sciences, University of Applied Sciences Magdeburg-Stendal, Stendal, Germany

**Keywords:** health equity, language barriers, mental health care, migrant health, multilingualism, professional communication, professional interpreters

## Abstract

A substantial number of people with mental disorders do not speak the language of their healthcare provider well enough to participate in a full diagnostic procedure or to conduct a cultural formulation interview. Five main strategies are used to overcome language discordance and provide multilingual mental healthcare: simplified direct communication, digital translation tools, multilingual materials, multilingual clinicians, and interpreters. Each has value, but each also has limitations. Informal interpreting by relatives, children, or untrained staff raises substantial ethical, clinical, and organizational concerns. Professional interpreting represents the preferred standard, yet access remains inconsistent in many health systems. This Perspective argues that multilingual communication should be treated as a core component of professional mental health care rather than as an exceptional or improvised accommodation. Therefore, mental health care services require multilevel action on individual, institutional and systemic levels to improve the quality of communication when patient and healthcare provider do not share the same language. The “Hamburg Declaration for Multilingual Healthcare” provides actionable guidance also for health professionals, health care management and healthcare users. Strengthening multilingual communication is not merely a technical improvement but a matter of clinical quality, patient safety, and health equity.

## 1 Introduction

### 1.1 Linguistic diversity

Linguistic diversity is a defining characteristic of contemporary societies. Approximately 6, 900 languages are actively used worldwide, and the 20 most widely spoken languages are spoken as first languages by 3.6 billion people1^1^. Governments regulate linguistic communication in different ways. Germany and Spain, for example, each have one official language at the national level, whereas Switzerland has four official languages and South Africa recognizes twelve.

Beyond official languages, many countries are characterized by considerable linguistic diversity. In Germany and Sweden, around 200 languages are spoken (1, 2). In the United States, more than 500 individual languages and language groups are spoken at home^2^. A country’s linguistic diversity reflects ethnic and linguistic heterogeneity as well as the size and composition of its migrant population.

Before migration, many migrants have limited opportunities to learn the language of the destination country. Language proficiency can improve substantially after arrival, but this depends on structural conditions such as access to language courses, different proficiency levels, stable funding, compatibility with employment and family responsibilities, and childcare provision (3). Even where language courses are available, barriers remain. In Germany, for example, 37% of migrants reported poor or no German language skills even after more than eleven years of residence (4).

Because public services, including administration, education, and healthcare, are usually provided in one or a few official languages, linguistic diversity can translate into unequal participation. This is particularly relevant in mental healthcare, where diagnosis and treatment rely heavily on verbal communication.

### 1.2 Language discordance in (mental) healthcare

Healthcare consultations are shaped by time pressure, power asymmetries, unfamiliarity with medical culture and terminology, and emotional distress (5). These demands are intensified when patients and providers do not share sufficient language proficiency. In mental healthcare, communication is especially central because diagnosis depends more strongly on verbal exchange than on physical examination or objective testing.

Empirical findings illustrate the scale of the problem. In the Netherlands, almost 40% of ethnic minority hospital patients have limited communicative ability in the dominant language (6). In psychiatric and oncological outpatient treatment in Germany, 12% of migrant patients did not speak German well enough for a regular consultation (7). In South Africa, more than 80% of medical consultations occur across communication barriers (8), and only 6% take place fully or partly in the patient’s mother tongue (9). In Romania, at least 13% of migrants reported limited access to health services due to communication barriers (10).

Language discordance in the diagnostic procedure and the conduction of the cultural formulation interview (CFI) can lead to serious negative consequences for the patient as well as for the mental health professionals. Refugees and migrants will be less likely to seek help in mental health services (11) when a stable communication is not offered. Conducting the CFI with their components like the explanatory models, the cultural identity, or perceived stressors and supports is very limited when a proper communication is not possible. Questions might not be understood, and answers might be kept short because of shame on behalf of the patient for not speaking well enough the official language. From the providers´ point of view, information might be missing or misunderstood and therefore a comprehensive diagnosis very difficult to achieve. On a more fundamental basis trust as the basis for the clinician-patient relationship is difficult if get supported when communication barriers exist (12).

It has proven that language discordance can contribute to poor adherence, and lower treatment success (13) as misdiagnosis, and premature discontinuation of treatment (14). Given that migrants may have elevated risks of mental health problems compared with native populations, communication barriers in mental healthcare require systematic attention (15).

## 2 Resources for overcoming communication barriers

Institutional, regional and national measures to promote multilingual healthcare depend to a large extent on the resources available and the policy framework. Health systems around the world vary greatly in terms of their resources and societal attitudes, which are reflected in the policy framework. Regardless of this, there are five main strategies that support multilingual communication in mental healthcare: adapted direct communication, technological aids, multilingual communication tools, multilingual practitioners, and interpreters.

### 2.1 Adapted direct communication

Direct communication between practitioner and patient may be possible even when they do not fully share the same language. For example, a patient may understand some English but feel less confident speaking it. Speaking slowly, using short sentences, and minimizing technical terminology can therefore improve communication. Mental health professionals can also encourage patients to express themselves by explicitly valuing even rudimentary language skills early in the conversation.

However, adapted direct communication has clear limits. It cannot replace professional language support in complex diagnostic assessments, psychotherapy, risk evaluation, informed consent, or emotionally sensitive conversations. Multilingual communication skills should therefore be integrated into the basic and continuing education of mental health professionals.

### 2.2 Technological aids

Technological aids designed to overcome cross-linguistic communication challenges barriers can facilitate communication in healthcare, particularly when professional interpreters or multilingual healthcare professionals are unavailable. In recent years, numerous applications have been developed or refined. Currently, 15 technological aids for overcoming communication barriers in healthcare are in use, including DeepL, iTranslate Converse, Universal Doctor Translator, Aidminutes, and Medi Babble (16). Google Translate is currently the most widely used application and supports 249 languages. For everyday use, such an easily accessible and free communication tool can be advantageous.

However, their use in mental healthcare requires caution. An evaluation of Google Translate for English-to-Arabic mental health information identified inaccurate and incomprehensible translations, incoherent phrasing, and critical errors (17). The use of AI tools in healthcare also raises serious data protection and confidentiality concerns, as sensitive patient data may be transmitted to third-party servers and potentially stored or reused (18).

Translation tools are regularly used by mental health professionals (19). Key concerns include not only limited translation accuracy and data protection risks, but also cultural inappropriateness, reduced empathy and trust, and poor workflow integration. Effective implementation is hindered by infrastructural constraints, institutional barriers, and the absence of clear policy frameworks. Overall, translation tools can be helpful for basic communication when registering for treatment or booking appointments, provided that data protection and confidentiality are fully guaranteed. For more complex and emotionally sensitive interactions like a diagnostic procedure, the informed consent, a medication plan or a psychotherapeutic treatment translation tools are not suitable (20).

Given the promise of AI-powered translation tools, it would be desirable for such tools to meet the requirements of the EU Medical Devices Regulation or comparable regulatory frameworks, particularly when used to overcome communication barriers in healthcare.

### 2.3 Multilingual communication material

Multilingual patient information can improve patients’ health literacy. Examples include multilingual guideline-based patient information and platforms such as Zanzu, which provides sexual health information in 13 languages, plain language, and sign language^3^. Beyond translation, cultural adaptation of patient information has been shown to be more effective than purely linguistic translation (21).

Healthcare institutions should provide key information in the languages most relevant to their patient populations. This includes websites, signage, forms, questionnaires, patient information, menus, and medication-related materials. Such work should be based on linguistic needs assessments and carried out by professional translators. Because materials require regular updating, professional translation services should be integrated into healthcare systems (22).

### 2.4 Multilingual practitioners

Patients with mental disorders often find communication easiest when they can speak with their practitioner in their first language. Multilingual practitioners therefore represent an important resource. In one German metropolitan outpatient mental healthcare context, 25 treatment languages were identified among 1,123 psychotherapists and psychiatrists; 30% used a language other than German in treatment, and 51% felt confident providing specialist treatment in another language (7).

Nevertheless, multilingual practitioners alone cannot meet all language needs. Their availability often does not match patient demand, and reliance on them may create disproportionate caseloads. It may also encourage monolingual practitioners to refer patients on the basis of assumed cultural familiarity rather than clinical need. Multilingual practitioners should be valued and supported, but they cannot replace systematic interpreter provision.

### 2.5 Interpreters

In healthcare, three main groups of interpreters are used. Their respective capacities and limitations are described below.

#### 2.5.1 Family members or friends

A common implicit attitude among many mental health professionals is: “If you want to understand me, bring someone to interpret.” This shifts responsibility for communication from the healthcare professional to the patient and can lead to omissions, distortions, filtering, and role conflicts. The use of children is particularly problematic. Children may lack the vocabulary and maturity required for medical communication, may be exposed to distressing information, and may experience disruption of family roles (23). For these reasons, the use of children as interpreters in healthcare should be rejected in order to protect their emotional well-being. Every healthcare facility should explicitly state this in its institutional rules.

Mental Health professionals in general cannot be certain that relevant information reaches the patient accurately when the accompanying person is not qualified to interpret.

When no professional interpreter is available and a layperson must assist, clinicians should act as conversation managers. They should brief the lay interpreter, clarify roles, maintain direct communication with the patient, and check understanding throughout the encounter.

#### 2.5.2 Multilingual staff

Multilingual staff are frequently used as *ad hoc* interpreters in healthcare settings. Some hospitals even maintain lists of staff who speak additional languages (24). However, this practice raises ethical, organizational, and legal concerns. Staff may be asked to interpret without training, quality assurance, remuneration, or contractual recognition. Interpreting duties may interrupt their primary work and create power imbalances between those requesting and those providing interpreting support (25).

For these reasons, multilingual staff should not be used as a routine substitute for professional interpreters. If no alternative exists, they should receive training, legal safeguards, and appropriate compensation.

#### 2.5.3 Professional interpreters

Professional interpreters represent the preferred standard for communication when patient and healthcare provider do not share the same language. In the United States, Section 1557 of the Affordable Care Act restricts the use of minors, family members, friends, and unqualified bilingual staff as interpreters in healthcare. Comparable safeguards are absent in many countries. According to the Migrant Integration Policy Index, only 10 of 56 countries provide qualified interpretation services free of charge for patients with insufficient proficiency in official languages^4^.

In many health systems, professional interpreting is therefore not structurally guaranteed. Individual professionals, institutions, or regions must develop their own solutions. Some hospitals integrate professional interpreting despite limited reimbursement, but such provision is less common in outpatient care. Telephone and video interpreting can improve access, especially in rural areas or for less common languages (26).

In many countries, uniform qualification standards for healthcare interpreters are lacking. Bottom-up initiatives have produced diverse roles and labels, such as community interpreters, cultural mediators, language mediators, refugee guides, and intercultural companions. Correspondingly, job profiles and training requirements vary widely (27). In Germany for example the level of qualification is rather low as community interpreters have completed an average of only 25 hours of training, while 30% have never attended any training. However, this is offset by the fact that payis often only slightly above minimum wage (28).

It would be target oriented if professional interpreters in mental healthcare would have sufficient language skills in the languages to be interpreted, knowledge about professional ethics, role perception, confidentiality, terminology and about interpreting techniques (27). Such a quality requirement can only be met if pay is significantly improved and appropriate training programmes are available nationwide.

Working with professional interpreters also requires specific skills from clinicians. Many healthcare professionals are unfamiliar with interpreters’ roles and responsibilities (29). Training in triadic communication^5^ can improve collaboration, role clarity, and clinical confidence (30). Alongside professionalism, empathy, teamwork, and pre- and post-session briefings, trust has been identified as an important relational quality in interpreter-mediated therapeutic relationships (12).

## 6 Toward professional standards

High-quality multilingual communication requires action at individual, institutional, regional, and national levels.

At the individual level, clinicians should understand limited shared language not as an exceptional problem but as a normal feature of diverse societies. This perspective encourages respectful communication, additional time where needed, and adaptation of communication style to patient needs.

At the institutional level, multilingual communication should be integrated into organizational policy. Patients’ preferred languages should be documented routinely. Key written materials should be translated into the most relevant patient languages. Institutions should define when professional interpreters are required, prohibit the use of children as interpreters, and provide staff training on multilingual communication. Multilingual professionals who are willing to provide interpreting support in addition to their regular duties should receive appropriate training and separate remuneration. Their services should be used only in clearly defined routine situations.

At the regional and national levels, quality standards for interpreting in healthcare and community settings should be implemented. Qualification programs should be accessible and publicly funded. Governments should provide sustainable funding for professional interpreting in mental healthcare. A long-term professionalization goal should be stable employment for interpreters rather than reliance on insecure freelance arrangements.

## 7 Discussion

Multilingual communication is often treated as an exceptional challenge in mental healthcare, although it is a predictable feature of diverse societies. This mismatch creates clinical, ethical, and organizational risks. Current reliance on informal interpreters, untrained bilingual staff, or unsupported clinicians is inconsistent with the professional standards expected in other areas of psychiatric care.

The central argument of this Perspective is that multilingual communication must become part of the infrastructure of mental healthcare. Adapted direct communication, digital tools, multilingual materials, multilingual clinicians, and interpreters all have a role, but they are not interchangeable. Professional interpreting should be available whenever communication barriers affect diagnosis, treatment, consent, safety, or the therapeutic relationship.

Future development should move beyond short-term projects toward stable implementation: funded interpreter services, national qualification standards, institutional multilingual policies, and systematic clinician training. In transcultural psychiatry, this is not a peripheral concern. It is central to equitable, safe, and person-centered care.

On a rather practical level the Hamburg Declaration for Multilingual Healthcare^6^ provides actionable guidance at the individual, institutional and systemic levels to improve the quality of communication when patient and healthcare provider do not share the same language.

## Data Availability

The original contributions presented in the study are included in the article/supplementary material. Further inquiries can be directed to the corresponding author.

## Footnotes

1 Ethnologue. How many languages are there in the world? Available at: https://www.ethnologue.com/insights/how-many-languages/

2 U.S. Census Bureau. Detailed languages spoken at home and ability to speak English for the population 5 years and over: 2017–2021. Available at: https://www.census.gov/data/tables/time-series/demo/language-use/2017-2021-lang-tables.html

3 Zanzu. Available at: https://www.zanzu.de/

4 MIPEX. Migrant Integration Policy Index 2025 – Health. Available at: https://mipex.eu/health-2025

5 Free training material are available under: www.ZwischenSprachen.de or www.trialog.inter-pret.ch

6 Declaration available under www.mim2m.net/declaration
